# Assessing the utility of large language models for phenotype-driven gene prioritization in the diagnosis of rare genetic disease

**DOI:** 10.1016/j.ajhg.2024.08.010

**Published:** 2024-09-09

**Authors:** Junyoung Kim, Kai Wang, Chunhua Weng, Cong Liu

**Affiliations:** 1 Department of Biomedical Informatics, Columbia University, New York, NY 10032, USA; 2 Raymond G. Perelman Center for Cellular and Molecular Therapeutics, Children’s Hospital of Philadelphia, Philadelphia, PA 19104, USA; 3 Department of Pathology and Laboratory Medicine, University of Pennsylvania, Philadelphia, PA 19104, USA

**Keywords:** large language model, rare disease diagnosis, gene prioritization, precision medicine, artificial intelligence, generative pre-trained transformers, phenotypes

## Abstract

Phenotype-driven gene prioritization is fundamental to diagnosing rare genetic disorders. While traditional approaches rely on curated knowledge graphs with phenotype-gene relations, recent advancements in large language models (LLMs) promise a streamlined text-to-gene solution. In this study, we evaluated five LLMs, including two generative pre-trained transformers (GPT) series and three Llama2 series, assessing their performance across task completeness, gene prediction accuracy, and adherence to required output structures. We conducted experiments, exploring various combinations of models, prompts, phenotypic input types, and task difficulty levels. Our findings revealed that the best-performed LLM, GPT-4, achieved an average accuracy of 17.0% in identifying diagnosed genes within the top 50 predictions, which still falls behind traditional tools. However, accuracy increased with the model size. Consistent results were observed over time, as shown in the dataset curated after 2023. Advanced techniques such as retrieval-augmented generation (RAG) and few-shot learning did not improve the accuracy. Sophisticated prompts were more likely to enhance task completeness, especially in smaller models. Conversely, complicated prompts tended to decrease output structure compliance rate. LLMs also achieved better-than-random prediction accuracy with free-text input, though performance was slightly lower than with standardized concept input. Bias analysis showed that highly cited genes, such as *BRCA1*, *TP53*, and *PTEN*, are more likely to be predicted. Our study provides valuable insights into integrating LLMs with genomic analysis, contributing to the ongoing discussion on their utilization in clinical workflows.

## Graphical abstract

**Figure:**
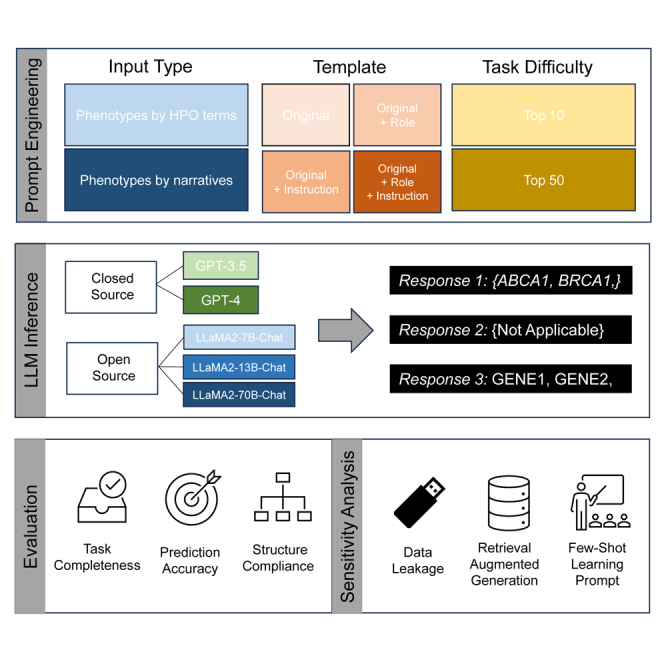

## Teaser

This study evaluates five large language models for phenotype-driven gene prioritization in rare disease diagnosis, focusing on task completeness, gene prediction accuracy, and output structure adherence. Despite improvements with larger models and sophisticated prompts, large language models, including GPT-4, still lag behind traditional tools, highlighting the need for further advancements.

## Introduction

Phenotype-driven gene prioritization is a process that involves identifying and ranking candidate diagnostic genes by examining an individual’s phenotypes. It plays a crucial role in rare disease diagnosis when analyzing genomic data from high-throughput (e.g., whole-genome/whole-exome sequencing) experiments or designing virtual sequencing panels for diagnosis purposes.^1^ The underlying premise is that disease-related phenotypes are caused by one or more gene dysfunctions. By leveraging the established genotype-phenotype disease associations, phenotype-based analytical models have been developed, resulting in numerous bioinformatics tools. For instance, Phenomizer^2^ compares individual phenotypes to a database of known genetic diseases and their associated phenotypes and ranks the potential genetic diseases based on the semantic similarity of the patient’s phenotype to known conditions within the Human Phenotype Ontology (HPO). Exomiser^3^ integrates various data sources, including gene-disease associations, variant databases, and Gene Ontology data, and employs a random-walk analysis to score and rank candidate genes or variants. Similarly, AMELIE^4^ constructed a comprehensive disease-phenotype knowledgebase by integrating various databases and resources, including ClinVar and Human Gene Mutation Database (HGMD), and parsing relationships from literature. It then developed a machine-learning classifier to rank candidate genes. Phenolyzer^5^ and its successor Phen2Gene^6^ integrated HPO annotations, gene-disease databases, and gene-gene networks and applied a probabilistic framework to build a phenotype-driven gene prioritization tool. Network inference methods based on modern deep-learning frameworks have also been explored. For example, DeepSVP^7^ constructs a graph from ontology axioms and employs the DL2Vec approach for gene-phenotype association prediction. CADA^8^ utilizes graph-embedding techniques to predict links between phenotype and diseases.

As introduced above, the majority of the existing tools rely on established knowledge databases or knowledge graphs that connect phenotypes with genes (or monogenic diseases). These databases are typically curated manually and informed by the literature, which can be ad hoc and lack scalability. Additionally, most of these bioinformatics tools can only process “term-based” input, requiring natural language-processing techniques to map terms from clinical notes to HPO concepts. We hypothesized that recent advancements in large language models (LLMs), trained on massive and diverse datasets, could potentially provide an end-to-end, text-to-gene solution to this task by leveraging their extraordinary ability to “understand natural language.”^9^^,^^10^ These models begin by employing a transformer-based decoder architecture to pre-train a base language completion model without supervision. Subsequently, the base model undergoes a fine-tuning process with human feedback and additional refinement through reinforcement learning, guided by a reward model trained using supervised methods, which can lead to the development of a ChatBot. Numerous articles have indicated the potential of LLMs for healthcare applications, including individual education,^11^ appointment scheduling,^12^ optimization of clinical decision support systems,^13^ aid in data collection for clinical studies,^14^ enhancement of information retrieval in electronic health records (EHRs),^15^ and summarization of evidence in publications.^16^

In this study, we will particularly focus on two advanced ChatBot-based LLMs: generative pre-trained transformer (GPT) series, including GPT-3.5 (also known as ChatGPT) and GPT-4 (https://www.mikecaptain.com/resources/pdf/GPT-1.pdf), and the Llama2 series, including Llama2-7b-chat (with 7b indicating 7 billion parameters), Llama2-13b-chat, and Llama2-70b-chat.^17^^,^^18^ We will explore the ability of standard, unmodified LLMs (vanilla LLMs) to analyze human phenotypic presentations and predict genetic diagnoses. Our primary objectives include evaluating LLMs’ performance in completing the designated task, achieving accurate gene prioritization, and adhering to output structure requirements. We will assess the impact of various factors, including prompts, model sizes, task difficulty levels, and phenotypic input type. Additionally, we will assess the impact of advanced methods like retrieval-augmented generation (RAG) and few-shot prompting on model performance and address data leakage concerns by repeating experiments with the same dataset at different times and using a new dataset gathered from post-2023 (Post2023) publications. Overall, this study provides a comprehensive assessment of how LLMs can potentially be integrated into the current clinical genomic analysis workflow for rare disease diagnosis.

## Material and methods

### Datasets

We utilized publicly accessible datasets consisting of a total of 276 de-identified individuals who had been diagnosed with monogenic Mendelian diseases,^6^ all with previously established diagnostic genes before 2021. There are a total of 165 distinct genes in the final pool of diagnosed genes. The cohort was pooled from five distinctive sources as outlined in Table 1, including a broad spectrum of Mendelian diseases and genes, ensuring that the dataset covered various genetic conditions. The TAF1 dataset only contains 14 individuals with variants in *TAF1* from one of the *American Journal of Human Genetics* (*AJHG*) articles.^19^ The Columbia_U dataset contains 27 (de-identified) individuals with 24 diagnostic genes from Columbia University Irving Medical Center. The Department of Genomic Diagnostics (DGD) dataset includes 85 (de-identified) individuals with 75 diagnostic genes from the DGD at the Children’s Hospital of Philadelphia. The CSH dataset comprises 72 (de-identified) individuals with 59 genes from Cold Spring Harbor (CSH) Molecular Case Studies articles. The AJHG dataset has 83 individuals with 13 genes from the *AJHG* articles. Except for the CSH and AJHG datasets (both were curated HPO from the Aho-Corasick algorithm embedded in Doc2HPO^20^), the other datasets were curated by doctors beforehand.^6^ An institutional review board (IRB) exemption was obtained from the Columbia University Review Board.

**Table 1:** Datasets used for LLM evaluation in this study

**Source**	**# of individuals with HPO terms as input**	**# of individuals with narratives as input**	**Average # of HPO (s.d.)**	**Average # of tokens (s.d.)**
AJHG	78	72	11.42 (6.43)	187.64 (88.87)
CSH	72	49	12.83 (8.30)	161.43 (102.38)
ColumbiaU	27	–	11.52 (4.73)	–
DGD	85	–	9.03 (4.06)	–
TAF1	14	–	34.86 (10.71)	–
Other	–	4	–	180.25 (143.98)
Post2023	–	130	–	270.09 (177.07)

The HPO concepts and final diagnosed genes of each individual were previously curated.^6^ Free-text phenotypic descriptions were further collected from 53 original articles for 125 individuals (including four individuals not included in the original HPO dataset for quality-control purposes). This was accomplished by identifying the relevant section in the articles and manually extracting the original text that provided detailed information about the individual’s phenotype presentations. One such example is as follows: “At 6 years, IQ was evaluated (verbal IQ 73, performance IQ 58). On physical examination at 15 years, she has a normal head circumference (+0 SD), facial dysmorphism, and thoracolumbar kyphoscoliosis.” To avoid data leakage, we manually excluded genetic/genomic analysis-related text from the original text. Additionally, we curated a new dataset of free-text phenotypic descriptions for 130 individuals from Post2023 publications by manually extracting the original text in the same way as described above. More details about the dataset can be found in the GitHub repository (https://github.com/stormliucong/LLM-Gene-Prioritization/tree/main/data/evaluation), including the reference information (if applicable) for the original articles from which we curated this dataset.

### Prompt engineering

To maximize the utilization of LLMs, effective prompt engineering is essential.^21^ A prompt refers to a set of instructions provided to an LLM to guide its response. Prompt engineering involves the process of designing and formulating prompts to elicit desired responses from language models.^22^ Considering that the fundamental function of an LLM is to accomplish language completion tasks, it is reasonable to assume that the quality, clarity, and specificity of the prompt could significantly impact an LLM’s performance.^23^ To assess how variations in prompts could influence the performance, we formulated various prompts based on the intricate interplay between roles, instructions, and model performance (as shown in Table 2): (1) the “original” prompt serves as the fundamental query to use individual phenotype features for gene prioritization. (2) The “original + role” prompt introduces a role assignment component, casting the model in the role of a “genetic counselor.” While preserving the core objective of gene prioritization, this prompt integrates a role-based perspective, which is also endorsed as a “system” message by OpenAI.^22^ (3) The “original + instruction” prompt includes additional guidance sentences for the model, instructing it to utilize its accumulated knowledge without requiring real-time database access for predictive purposes. (4) The “original + role + instruction” prompt integrates the role of a genetic counselor with the previously mentioned instruction, offering the most comprehensive approach. Each input prompt was presented as a zero-shot^24^ scenario without providing examples (except for output structure examples). Besides different prompt engineering techniques, we also considered different factors in constructing the final prompt. We assessed two input categories for individual phenotypic features: those presented through HPO terms and those via free-text phenotypic descriptions (obtained as described above). In addition, we designed an easy task (i.e., making a correct gene prediction within the top 50 predictions) and a challenging (i.e., within the top 10) task to evaluate the performance.

**Table 2:** Prompt engineering involved for phenotypic-based gene prioritization task

**Prompt template**	**Prompt content**^a^
Original	The phenotype description of the patient is [phenotype list]. Can you suggest a list of [top k] possible genes to test? Please return gene symbols as a comma separated list. Example: “*ABC1*, *BRAC2*, *BRAC1*” or “not applicable” if you cannot provide the result.
Original + role	Consider you are a genetic counselor. The phenotype description of the patient is [phenotype list]. Can you suggest a list of [top k] possible genes to test? Please return gene symbols as a comma separated list. Example: “*ABC1*, *BRAC2*, *BRAC1*” or “not applicable” if you cannot provide the result.
Original + instruction	The phenotype description of the patient is [phenotype list]. Can you suggest a list of [top k] possible genes to test? Please use the knowledge you have trained. No need to access to the real-time database to generate outcomes. Also, please return gene symbols as a comma separated list. Example: “*ABC1*, *BRAC2*, *BRAC1*” or “not applicable” if you cannot provide the result.
Original + role + instruction	Consider you are a genetic counselor. The phenotype description of the patient is [phenotype list]. Can you suggest a list of [top k] possible genes to test? Please use the knowledge you have trained. There is no need to access the real-time database to generate outcomes. Also, please return gene symbols as a comma separated list. Example: “*ABC1*, *BRAC2*, *BRAC1*” or “not applicable” if you cannot provide the result.
Few-shot prompts	Instruction: Question: consider you are a genetic counselor. Given the phenotype description of the patient listed above. Suggest the top [top k] genes to test. Provide the gene symbols in a “comma-separated list” or “not applicable” if no relevant genes are identified. Examples: Phenotype: neoplasm Response: *PTEN*, *BRCA1*, *BRCA2*, *TP53*, *KRAS*, *EGFR*, *MYC*, *PALB1*, *RB1*, *VHL* Phenotype: seizure, hypotonia, global developmental delay, abnormal facial shape, microcephaly, mandibular prognathia, severe global developmental delay Response: *SCN1A*, *MECP2*, *CDKL5*, *KCNQ2*, *STXBP1*, *ARX*, *FOXG1*, *PCDH19*, *TCF4*, *MEF2C* Phenotype: hello, world, Earth, peace, love, toy, hamburger, salad, apple, Google, smoke, drug Response: not applicable Phenotype: Response: not applicable – Please answer: Phenotype: [phenotype list] Response:

a [Phenotype list] can be either a set of HPO-based concept names separated by “;” or a narrative containing phenotype descriptions extracted from the original literature. [top k] can be either “top 10” or “top 50,” representing challenge and easy tasks, respectively.

### Experiment design

For the GPT series, we utilized the OpenAI application programming interface (API), making API requests with various prompts to retrieve the gene prioritization predictions for each prompt. GPT-3.5-turbo was used for GPT-3.5 evaluation. We set the temperature parameter to zero to make the predictions more deterministic. In total, we conducted 32 experiments by considering possible combinations of various factors for each individual, multiplying two GPT versions (GPT-4 and GPT-3.5), four types of prompts (original, original + role, original + instruction, and original + role + instruction), two phenotypic features input types (HPO concepts and free text), and two predictive task thresholds (top 10 and top 50).

For the Llama2 series, we utilized Replicate’s API (a cloud-based service that provides access to a wide range of pre-built models) to assess the three Llama2-chat models with different parameter sizes. To save study costs, we used only the most complicated prompts (original + role + instruction) in the experiments. In total, there were 12 experiments for each input case by multiplying three Llama2-chat versions (7b, 13b, 70b), two phenotypic features input types, and two predictive task thresholds.

All experiments were iterated three times (i.e., three LLM responses generated independently with the same input) to measure the variability of LLMs. A previous report^25^ demonstrated the possible time-dependent nature of GPTs. To reduce the bias associated with calendar days and because of the closed-source nature of GPT, we permutated the experiment sequence for the GPT series. The final OpenAPI execution date was in August 2023 (except for the sensitivity analysis as described below). For the Llama2 series, we did not have that concern due to its open-source nature.

### Evaluation metrics

We assessed the performance of the LLMs on three outcomes, including the task completeness of the output, accuracy of generated gene lists, and adherence to specified output structural requirements. The evaluation metrics were specified as follows.

Task completeness measures whether LLMs can produce a “true” gene list for gene prioritization tasks. This metric assesses whether GPT behaves like a layperson, understanding the question and attempting to complete the task meaningfully. More specifically, if fewer than half of the expected number of genes were returned (e.g., <25 out of the top 50 predictions), we considered that LLMs did not complete the task. It’s important to note that we excluded fabricated genes and counted duplicated genes in the prediction list only once. If LLMs declined to produce prediction results, we also considered it as an incomplete task. Figure S1 presents two instances of LLM responses indicating task incompletions.

Accuracy of gene predictions (or Precision@K) evaluates whether the true diagnosed gene is identified within the top (10 or 50) predicted gene list generated by LLMs. Two sub-metrics were designed. The first is completed accuracy, which considers only experiments that completed the task as mentioned above for accuracy assessment. The second is overall accuracy, which includes all experiments. For experiments where the tasks were not completed, we considered the gene prediction results to be incorrect for the overall accuracy calculation. Figure S2 presents examples of correct or incorrect predictions made by an LLM.

Output structure compliance evaluates whether LLMs’ responses adhere to the specified output format requirement. Given an LLM’s tendency to provide free-text responses, we deemed the outcome compliant if a portion of the response matched a set of predefined regex patterns. As we instructed the LLM to generate “[not applicable]” for tasks that could not be completed, we could independently evaluate this metric regardless of task completeness. Figure S3 presents examples of an LLM’s output being non-compliant with the output structure instruction. This metric is designed to assess whether LLMs can be seamlessly integrated into an informatics pipeline.

For each experiment e, we calculated the task completeness rate (or gene prediction accuracy rate, output structure compliance rate) $p_{e}^{\left(t\right)}=\sum_{m=1}^{M}\frac{I^{\left(t\right)}\left(m,e\right)}{M}$ for each repetition t(t=1,2,3), where M is the total number of samples for evaluation in this experiment (for completed accuracy, M is the total number of samples completed in the task), and $I^{\left(t\right)}\left(m,e\right)$ = 1 if the task was completed (or gene prediction is accurate, output structure is compliant). For each metric, we then calculated the average outcome by averaging over three iterations. To provide more information about the precision and reliability, we also calculated 95% confidence intervals (95% CIs) for the average outcome using a bootstrap approach with replacement over 100 iterations.

Due to the substantial number of experiments and the significant human effort required to assess the outcomes, we developed a script to automate the measurement of the three aforementioned metrics. To assess whether a task was completed, we parsed the responses and compared them against HUGO Gene Nomenclature Committee (HGNC) gene symbols (including previous symbols and alias symbols) using regular expression while excluding common gene name errors (e.g., *SEPT1*, *MAR1*) as detailed in the Gene Name Errors Screen project.^26^ This script was also used to measure the accuracy of gene predictions by comparing the responses with the diagnosed gene HGNC symbols (including previous symbols and alias symbols). To evaluate adherence to the output format, we established regular expression patterns based on the prompt’s requirements and searched for these patterns within LLMs’ responses. The output of this script was inspected manually on 250 randomly selected experiments, and 100% accuracy was achieved.

### Sensitivity analysis

To enhance our understanding of factors affecting LLM performance, we conducted sensitivity analyses. We tested few-shot learning prompts, a method where the model is provided with a small number of example prompts and responses to guide its predictions, which has been shown to improve LLM performance.^27^^,^^28^ We aimed to evaluate the effectiveness of this approach by comparing the top 10 gene predictions from few-shot prompts (Table 2; few-shot prompts) with the original + role + instruction zero-shot prompt using HPO concepts in the GPT series.

Previous studies have revealed that ChatGPT might suffer from data contamination on the evaluation benchmark.^29^ To address concerns about potential data leakage, we compiled a new dataset (Table 1; Post2023) from Post2023 publications. This involved searching genes from the final diagnosed pool in the original set and randomly collecting cases from case reports. Both the new dataset and the original were analyzed/re-analyzed during the same week in June 2024.

Additionally, we employed RAG using the LlamaIndex library (https://github.com/jerryjliu/llama_index).^30^^,^^31^ This technique begins by indexing a collection of documents where each document is associated with a gene or a phenotype. The index is built on LLM-generated embeddings, allowing us to query relevant documents based on their similarity to a given prompt. For indexing, we used two approaches, both structured around HPO annotations. (1) The first is gene-to-phenotype (G2P): each document in this index associates a specific gene with a list of phenotypes. The documents are structured to contain a narrative such as “The phenotypes associated with gene [gene_name] include [list of phenotypes].” (2) The second is phenotype-to-gene indexing (P2G): conversely, in this approach, each document lists genes associated with a specific phenotype. We employed two different sizes of text embeddings (text-embedding-3-large and text-embedding-3-small) provided by OpenAI to create the indices. The top 10 similar documents were retrieved for GPT-4 response generation using the original + role + instruction prompt. The experiments were executed in June 2024.

## Results

### Accuracy of gene predictions among different models

**Figure 1:**
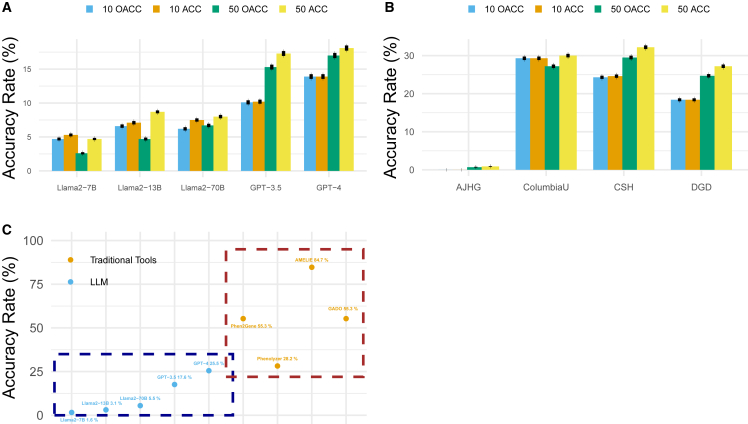
Gene prediction accuracy across different LLMs and datasets (A) Accuracy rate across different LLMs with original + role + instruction prompt. Error bars represent standard deviation. (B) Prediction accuracy across different datasets for GPT-4; TAF1 datasat was excluded because all the prediction accuracy was 0. Error bars represent standard deviation. (C) Comparison between LLMs against other bioinformatics tools in predicting top 50 candidate genes in the DGD dataset. ACC, accuracy, defined as the proportion of correct predictions out of the total predictions made in completed experiments; OACC, overall accuracy, which includes all experiments. If the tasks were not completed, overall prediction accuracy results were considered incorrect.

Figure 1A shows the overall prediction accuracy rate of LLMs when utilizing the original + role + instruction prompt. On average, GPT-4 achieved the highest overall prediction accuracy rate of 13.9% (95% CI: 11.73%–15.85%) and 17% (95% CI: 14.59%–19.16%) for the top 10 and top 50 tasks, respectively, followed by GPT-3.5 (10.1%, 95% CI: 7.99%–11.78%; 15.3%, 95% CI: 13.33%–17.18%), Llama2-70b-chat (6.2%, 95% CI: 4.38%–7.70%; 6.7%, 95% CI: 5.15%–8.13%), Llama2-13b-chat (6.6%, 95% CI: 5.13%–8.02%; 4.7%, 95% CI: 3.37%–5.90%), and Llama2-7b-chat (4.7%, 95% CI: 3.45%–5.76%; 2.6%, 95% CI: 1.24%–3.50%). The accuracy rate among completed tasks showed a similar trend but with smaller gaps among different models. Figure 1B further demonstrates GPT-4’s accuracy among different datasets for the top 50 tasks. As the best-performed LLM, GPT-4 displayed the highest average overall accuracy rate of 29.51% (32.24% among completed) in CSH, closely followed by ColumbiaU (27.16%, 30.03% among completed). In the context of the top 10 tasks, GPT-4 performed best in ColumbiaU, with CSH in close pursuit. The performance in DGD was noted at 24.71% (27.21% among completed), while the performance in AJHG was only 0.75% (0.89% among completed), and TAF1 exhibited the lowest rate of 0% (not shown in the figure) because *TAF1* was never predicted by GPTs. This result deviates from the trends observed in the results from other tools, where TAF1 typically showed the best performance with close to perfect accuracy, followed by ColumbiaU, DGD, AJHG, and CSH. In general, even the best-performing LLM still lags behind traditional bioinformatics tools. For instance, the best-performing GPT model demonstrated an average accuracy rate of 26.91% in the CSH dataset (24.31% for top 10 and 29.51% for top 50), which is lower than Phen2Gene’s accuracy (35.3% for top 10 and 55.3% for top 50 on the same CSH dataset).^6^ Other methods, such as Phenolyzer and AMELIE (using only HPO), also showed approximately 40% accuracy for the top 50 prediction,^32^ outperforming the best GPT models. Additionally, “real-world” accuracy can be even lower because LLMs cannot always guarantee task completion or proper output formatting, which can be automated in a workflow. An example of comparing performance between LLMs and traditional bioinformatics tools was shown in Figure 1C, where the accuracy of predicting the top 50 candidate genes in the DGD datasets is plotted.

### Gene-dependent prediction bias analysis

**Figure 2:**
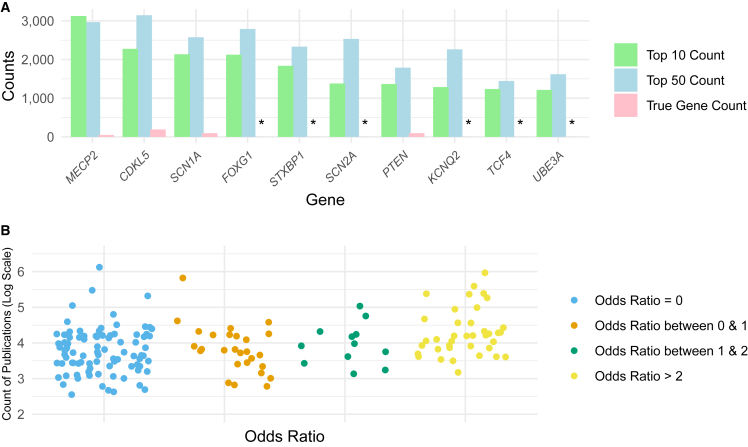
Gene-dependent bias analysis (A) Genes most frequently predicted by LLMs across all experiments. The green bar represents the number of times each gene was predicted in the challenging task (top 10), the blue bar shows the number of times each gene was predicted in the easier task (top 50), and the red bar indicates the number of times each gene appeared in the final diagnosed gene pool (i.e., ground truth occurrence). An asterisk (^∗^) denotes genes that never appeared in the final diagnosed gene pool, indicating a high false-positive potential of LLMs for these genes. (B) Correlation between Google Scholar counts and the odds ratio, which is calculated as the observed prediction times versus expected prediction times for truly diagnosed genes across different models for all experiments. The correlation between log-scaled Google Scholar count and odds ratio is categorized into four groups.

A further investigation of the LLM’s response revealed potential gene-dependent bias associated with the LLMs, which might explain the variations in accuracy observed across datasets. Figure 2A shows the top 10 genes most frequently predicted by GPT-4 across all experiments, with six of them not appearing in the diagnosed gene pool. For example, *FOXG1* consistently appeared in GPT-4’s output despite never occurring in the diagnosed pool, with 2,125 experiments (22.1%) in the top 10 predictions and 2,791 experiments (29%) within the top 50 predictions. Overall, 6,383 genes never occurred in the final diagnosed pool but were predicted by at least one of the LLMs. In contrast, genes that frequently appeared in the diagnosed gene pool, such as *DHX30* (previously "*DDX30*" in HGNC nomenclature), were notably absent from both the top 10 and top 50 predictions among all experiments from all LLMs. Table S1 shows the odds ratio calculated as the observed prediction times versus expected prediction times for truly diagnosed genes across different models for all experiments. Furthermore, considering the 165 genes in the final diagnosed pool given the original + role + instruction prompt, GPT-4 did not predict 88 genes (OR = 0), and GPT-3.5 failed to predict 107 genes. Llama2-70b-chat missed predictions for 135 genes, Llama2-13b-chat for 140 genes, and Llama2-7b-chat for 151 genes. A deeper investigation revealed that genes overpredicted by LLMs received substantial attention in research studies, with a higher number of Google Scholar search results. Figure 2B illustrates the correlation between the count of publications on a log scale and the odds ratio. As the count of publications increases, the odds ratio tends to increase (i.e., more likely being predicted). For genes that were not predicted accurately, the number of Google search (as of April 2024) hits was often less than 10,000 (e.g., *TAF1*: 9,950; *DHX30*: 1,010; *WDR26*: 972). In contrast, genes that were predicted with high accuracy had a significantly larger number of search hits (e.g., *BRCA1*: 471,000; *TP53*: 424,000; *PTEN*: 673,000), except for *CDKL5* (8,350), which had fewer cases than *TAF1* but still exhibited high accuracy in some LLMs.

### Effect of different factors in predicting accuracy

Tables S2–S5 show the gene prediction accuracy rate under different settings in GPTs and Llama2 series for the top 10 and top 50 prediction tasks, respectively. As expected, the average gene prediction accuracy for the more challenging task (i.e., making correct predictions within the top 10) was lower (12.11%, 95% CI: 11.30%–12.97%; 10.15% overall, 95% CI: 9.69%–10.78%) than the easier task (17.41%, 95% CI: 16.43%–18.32%; 13.06% overall, 95% CI: 12.43%–13.80%). However, the differences between these two tasks do not seem as large as expected. In fact, the Llama2-7b-chat and Llama2-13b-chat models displayed the opposite trend, especially for the overall accuracy, which might be due to the large number of samples that did not complete the top 50 tasks. These results could indicate that LLMs may either make correct predictions early in the sequence of output or cannot make correct predictions at all.

In the top 50 tasks (Tables S4 and S5), HPO concept inputs achieved an average accuracy rate of 20.67% (95% CI: 19.03%–22.24%) for GPT-4 (18.18% overall; 95% CI: 17.21%–19.60%), whereas free-text inputs had an average accuracy rate of 12.52% (95% CI: 9.95%–14.15%) (11.67% overall; 95% CI: 9.72%–12.98%). GPT-3.5 and Llama2-series showed the same tendency. A similar trend was observed in the top 10 tasks but with less discrepancy (Tables S2 and S3), suggesting that despite LLMs’ ability to understand narratives, structured input can lead to better prediction accuracy.

We did not observe a significant effect of prompts on the accuracy of gene predictions in GPT-4. However, we found that the prompt matters in GPT-3.5, especially for overall accuracy, where more detailed prompts generally yield better accuracy. The original prompt yielded 2.41% (95% CI: 1.64%–3.55%) overall accuracy in predicting the top 50 genes, while the original + role + instruction prompt showed 15.3% (95% CI: 13.00%–16.85%) (Table S4), which can be explained by the significant impact of prompts on the task completion rate observed in GPT-3.5 (described in the section below). A similar trend was observed in the predictions of the top 10 genes, albeit with less discrepancy.

### Task completeness among different models

The overall average task completion rate was 78.34%, with the average for GPT models at 79.42% (95% CI: 78.95%–79.96%) and for Llama2 models (using the original + role + instruction prompt) at 75.45% (95% CI: 74.66%–76.28%). Similar to the accuracy rate, larger models tended to achieve higher task completion rates. Specifically, GPT-4 achieved an almost perfect average completion rate of 94.22% (95% CI: 93.74%–94.82%) across all scenarios, which decreased to 64.62% (95% CI: 63.54%–65.66%) for GPT-3.5. This trend was also observed in the Llama2 series (using the original + role + instruction prompt only), where the 7b-chat model had a completion rate of 70.70% (95% CI: 68.65%–72.41%), the 13b-chat model 72.82% (95% CI: 71.20%–75.03%), and the 70b-chat model 82.83% (95% CI: 81.31%–84.34%).

The prompts significantly influenced GPT-3.5. As shown in Table S6, the average completion rate for GPT-3.5 was 23.15% (95% CI: 20.96%–24.78%) with the simplest prompt, increasing to 93.93% (95% CI: 93.14%–94.87%) with the most complex prompt.

All LLMs were more likely to complete easier tasks except the Llama2-70b-chat model. When requesting only the top 10 genes, an average completion rate of 83.82% (GPT-4, 98.86%; GPT-3.5, 68.79%; Llama2-7b-chat, 87.11%; Llama2-13b-chat, 91.85%; Llama2-70b-chat, 82.46%) was achieved. However, extending the request to include the top 50 responses resulted in a lower rate of 75.02% (GPT-4, 89.59%; GPT-3.5, 60.45%; Llama2-7b-chat, 54.28%; Llama2-13b-chat, 53.78%; Llama2-70b-chat, 83.21%) (Table S6). Further investigation revealed that the largest discrepancies occurred when GPT-3.5 was tasked with generating the top 50 results with the original prompt and free-text input, showing a 31.73% difference in average task completion rates compared to the same setting with the top 10 generating task (top 10, 52.00%; top 50, 20.27%) (Table S8). One possible explanation is that GPT-3.5 may undergo post-release alignment^16^ to adhere to ethical guidelines (https://openai.com/index/openai-safety-update/). This alignment could lead it to refuse to answer certain questions, including those related to politics^33^ or medicine.

Similarly, input type also had an impact. Across the models, GPT-4 models generally had higher completion rates on free-text input; for GPT-4, it ranged from 93.40% (95% CI: 92.96%–93.99%) for HPO to 96.03% (95% CI: 95.38%–96.65%) for free-text, and for GPT-3.5, rates ranged from 62.79% (95% CI: 61.59%–64.04%) to 68.67% (95% CI: 66.78%–69.93%). However, two out of three Llama2-chat models had higher completion rates with HPO input: Llama2-7b-chat achieved 71.56% (95% CI: 69.48%–73.41%) with HPO and 68.80% (95% CI: 64.40%–72.99%) with free text, while Llama2-70b-chat had 85.27% (95% CI: 83.40%–87.08%) with HPO and 77.47% (95% CI: 74.62%–81.45%) with free text. Conversely, Llama2-13b-chat showed the opposite trend, with 70.89% (95% CI: 68.41%–73.07%) for HPO and 77.07% (95% CI: 73.68%–80.17%) for free text (Table S6).

### Structure compliance

Table S7 demonstrates the output structure compliance rate for various experiments. Across nearly all settings, GPT-3.5 struggled to generate compliant output responses, achieving an average compliance rate of 27.32% (95% CI: 26.40%–28.29%). Llama2 models almost did not generate any compliant responses. Interestingly, in contrast to the task-completeness assessment, the original prompt achieved the highest average compliance rate of 62.97% (95% CI: 60.72%–65.22%) in GPT-3.5, while additional prompts significantly decreased output structure compliance to 30.59% (95% CI: 29.08%–32.40%) with original + role, 15.46% (95% CI: 13.81%–16.88%) with original + instruction, and 0.25% (95% CI: 0%–0.48%) with original + role + instruction. In contrast, GPT-4 exhibited a significantly higher and more robust output structure compliance rate with an average of 79.28% (95% CI: 78.49%–80.07%). Furthermore, the task difficulty level was crucial; in GPT-4, the top 10 tasks achieved 99.98% (95% CI: 99.96%–100.00%), while the top 50 achieved 58.58% (95% CI: 57.28%–60.27%). This trend was similarly observed in GPT-3.5, albeit with a smaller discrepancy of 31.17% (95% CI: 29.63%–32.42%) for the top 10 and 23.46% (95% CI: 22.32%–24.65%) for the top 50. Input types also impacted compliance, with HPO inputs achieving an average compliance rate of 80.01% (95% CI: 79.00%–80.83%) with GPT-4 compared to 77.67% (95% CI: 76.20%–78.99%) for free-text inputs. A similar trend was observed in GPT-3.5.

### Performance based on sensitivity analysis

**Figure 3:**
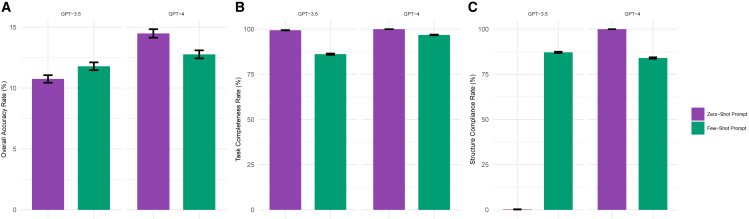
Performance comparison for zero-shot and few-shot prompts across GPT-3.5 and GPT-4 models (A) Overall accuracy rate across different versions of GPT models with different prompts based on HPO concepts input and top 10 experiment results. Error bars represent standard deviation. (B) Task completeness rate across different versions of GPT models with different prompts based on HPO concepts input and top 10 experiment results. Error bars represent standard deviation. (C) Structural compliance rate across different versions of GPT models with different prompts based on HPO concepts input and top 10 experiment results. Error bars represent standard deviation.

Figure 3 compares the performance of zero-shot (with the original + role + instruction prompt) and few-shot learning under the same settings (i.e., top 10 and HPO-based input). While few-shot learning slightly improved overall accuracy (Figure 3A) in GPT-3.5, it reduced the accuracy in GPT-4. It also did not help in completing tasks (Figure 3B). Notably, the overall accuracy gains in GPT-3.5 were mainly attributed to the enhanced structure compliance, which averaged only 0.24% in zero-shot learning scenarios (95% CI: 0%–0.48%) but escalated to 87.18% (95% CI: 82.15%–93.04%) with few-shot learning (Figure 3C).

**Figure 4:**
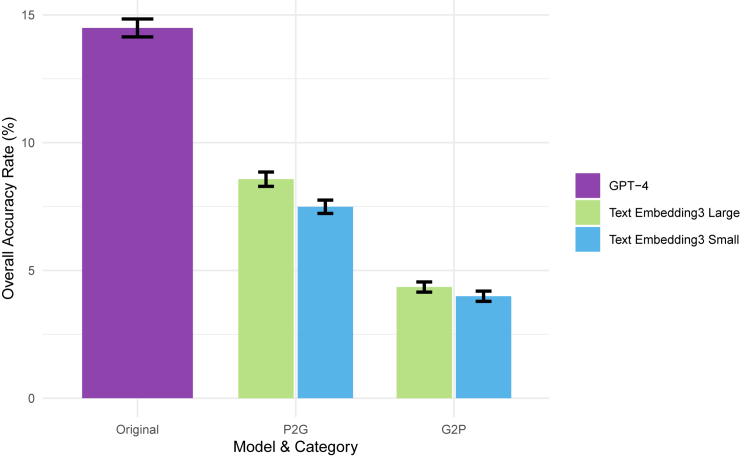
Overall accuracy rate for different embedding models applied in RAG methods Overall accuracy rate across GPT-4 model with different text embedding models based on HPO concepts input and top 10 experiment results. Error bars represent standard deviation.

Figure 4 compares different RAG approaches with the original experiment results under the same settings (i.e., top 10 and HPO-based input with the original + role + instruction prompt). RAG involves retrieving relevant information from a database and using this information to generate responses, effectively updating the prompt with relevant data. The original method makes predictions without any prior examples, relying solely on the model’s pre-trained knowledge and the provided prompt. Performance decreased significantly with RAG methods, but the P2G approach significantly outperformed the G2P method with an average accuracy gain of 3.8%. In both P2G and G2P methods, larger embeddings consistently show better accuracy, with an increase of 0.4%–1.1%.

**Figure 5:**
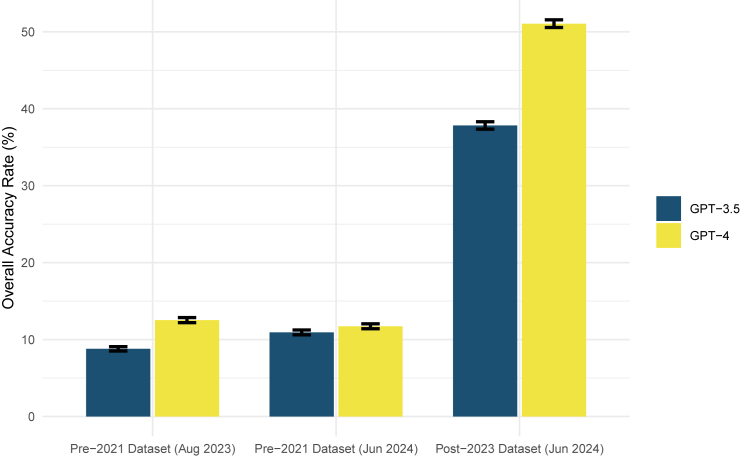
Overall accuracy rate across different GPT 3.5 and GPT-4 models using datasets from various time periods Overall accuracy rate performance with different datasets categorized by collection periods. Error bars represent standard deviation.

Figure 5 compares the overall accuracy rate among three sets of experiments under the same settings (free-text-based, top 10 predictions only): (1) the original dataset (cases prior to 2021) with experiments executed in August 2023, (2) in June 2024, and (3) a new dataset (Post2023), with experiments executed in June 2024. The differences between experiments using the same dataset in different months are small. However, the experiment with the new dataset in June 2024 showed a higher accuracy rate of 44.44% (95% CI: 41.38%–48.59%). A possible explanation for this performance gain could be the biased collection of this new dataset, where relevant articles are identified through Google searches of gene names, potentially increasing the inclusion of highly cited genes in this dataset. As an unbiased analysis, Figure S4 provides a gene-level comparison for those overlapped in both datasets. These findings indicate that, for this specific task, contamination in GPTs is not a significant concern when evaluating performance.

### Stability analysis of GPT

We investigated the stability of GPT-generated responses. By combining all experiments, including sensitivity analysis, there are 10,671 unique experiments. Of these, 1,166 (10.9%; 13.0% of those completed the task in at least one iteration) yielded different completion results across three iterations. In terms of overall accuracy, 341 (3.2%; 27.5% of those making accurate predictions in at least one iteration) arrived at different accuracies across the three iterations. Regarding structure compliance, among the 5,296 experiments, 1,120 (13.3%; 24.7% of those yielding compliant results) exhibited differences in compliance across the three iterations. Tables S9–S11 display the variability in outcome ratios across three iterations, breaking down the contributing factors. Few-shot learning demonstrates higher variability for all outcomes.

We further explored the impact of different calendar months on GPT variants by conducting identical sets of experiments in August 2023 and June 2024. Out of 250 unique experiments, 211 maintained the same number of completed iterations in both sessions. Meanwhile, 21 experiments showed changes from all iterations being completed in three attempts to none completed or vice versa. Regarding accuracy, 240 experiments maintained the same accuracy, while five experiments showed changes. For structural compliance, however, the figures were 125 versus 118, suggesting potential calendar month impact on GPT’s output structure.

## Discussion

Previous studies have consistently shown that LLMs achieved remarkable performance across various medical applications, including the ophthalmology exam,^34^ USMLE sample exam,^35^ and progress notes summarization.^36^ A few studies have explored the application of LLMs in the clinical genetic/genomic fields.^27^^,^^37^^,^^38^ Our results show current LLM-based predictions did not match the performance of software specifically designed for this task. Despite their ability to understand free-text input, LLMs generally achieved better predictions when using human-curated HPO concepts instead of free-text inputs. Accuracy improves with larger model sizes, and sophisticated prompts enhance task completeness, especially in smaller models, though they may reduce output structure compliance. Bias analysis revealed that LLMs more frequently predict highly cited genes, such as those related to cancer (e.g., *BRCA1*, *TP53*, and *PTEN*).

Base LLMs were initially designed for next-token prediction,^39^ a task where the model predicts the next word or token in a sequence based on the preceding context. ChatBot-like models are required to perform this task without extra fine-tuning. GPT-3.5 trained similarly to InstructGPT and optimized for dialog from GPT-3 (https://openai.com/index/chatgpt/). Llama2-chat is also fine-tuned for dialog usage based on Llama2 starting from supervised fine-tuning (SFT) and then utilizing reinforcement learning with human feedback (RLHF).^17^ GPT-4 also utilized RLHF and supports a multimodal model.^40^ When we used the base Llama2 model, it lacked comprehension and generated text identical to the questions and examples mentioned in the prompts (specific prompts were designed with “answer is:” pending as the last few tokens to prompt base models for the next token prediction task). This tendency persisted regardless of the number of genes to be generated or variations in phenotypic descriptions; it always begins with a gene provided in the prompt example (in this case, *ABCA1* or *ABC1*) until a specified token length was satisfied. The accuracy of gene prediction by Llama2 without being fine-tuned for dialog was entirely random. However, once the model is fine-tuned for conversation (GPT-3.5/GPT-4 or Llama2-chat), it can provide better-than-random guesses for gene prediction without further fine-tuning for clinical tasks.

Additionally, we identified instances of hallucinations generated by all models, particularly in the top 50 predictions. These occurrences were often associated with generating spurious gene symbols within a gene family. For example, starting from *OPA1*, a real gene, GPT-3.5 extends to *OPA50* in sequence, which are not actual genes. Previous studies have highlighted the same limitations in answering medical questions^41^^,^^42^ by attaching fabricated references. This deficiency may explain why GPT sometimes generates fictitious gene names when dealing with gene symbols, as gene symbols can function as both acronyms and identifiers. One potential explanation is that the byte pair-encoding (BPE) tokenizer, employed by both GPT and Llama2, breaks down references or gene symbols, allowing GPT to fabricate references or gene symbols using partial segments.

Our findings have a few implications. First, while current LLMs might excel at tasks that laypeople can handle, their performance does not match that of specialized tools for tasks requiring expert knowledge and models trained on specialized knowledge bases. To enhance prediction accuracy, it is necessary to fine-tune models with specialized datasets or knowledge resources, as seen in other tasks.^43^^,^^44^ Studies have shown that in differential diagnosis tasks, the Med-PaLM2, which was fine-tuned with medical domain data based on the LLM PaLM2,^45^ outperformed regular GPT-4 in prediction accuracy by approximately 10% and even exceeded clinician performance.^46^ Second, we may still want to utilize HPO or other ontologies as intermediaries in the era of LLMs. Even though machines can understand unstructured natural language, they still perform better with encoded and structured phenotype concepts. Alternatively, prompts should be designed to break down tasks further when the input is a natural language description, providing better guidance to LLMs through a two-step approach, such as chain-of-thought prompting. Lastly, general-purpose LLMs are more susceptible to “common attention” bias. This is particularly significant for rare disease diagnosis, as it suggests that general-purpose LLMs like GPTs may be more inclined to predict commonly encountered cases but may not perform as effectively with rare conditions. Similar conclusions were drawn from the differential diagnosis tasks, where clinicians noted that LLMs were useful for simple cases but had limitations for complex cases, focusing on specific aspects rather than holistic assessments.^46^

Nevertheless, the future seems promising. We consistently observe that larger models achieve better results across almost all evaluation metrics. This aligns with previous studies that reported a positive correlation between model size and performance. For example, GPT-4 outperformed GPT-3.5 in biomedical classification tasks, reasoning tasks,^47^ and question-answering accuracy.^16^ A similar pattern was observed in Llama2 models.^48^ The parameter size of GPT-3.5 and GPT-4 is approximately hundreds of billions to trillions, which is larger than the 7 billion, 13 billion, and 70 billion parameters in the Llama2 series. While GPT-4 achieved the best performance in all metrics, Llama2-70b-chat also outperformed its smaller counterparts. This implies that even larger LLMs might eventually match or surpass traditional knowledge graph-based approaches. However, a plateau effect might also exist. Given the rapid release of numerous pretrained LLMs, it’s challenging to include and keep up with all the latest models. Moving forward, establishing and maintaining an updated benchmark registry for various real-world clinical tasks is crucial. This registry would showcase LLM performance, empowering informed decision-making.

Previous studies have suggested that the variability in results affected performance metrics when classifying functional evidence in biomedical literature.^49^ Another study on a biomedical semantic question and answer task, repeated five times with the same model, concluded that despite the variability, its impact was minimal.^50^ In our evaluation of overall accuracy, despite larger variabilities compared to experiments repeated within the same month, the variation in model performance generally remained within acceptable parameters. However, significant variability was observed in structure compliance metrics across different months. More investigation is needed to understand how these variabilities impact downstream applications.

While studies have shown that expanding GPT with more examples (few-shot learnings) and using retrieved instructions (RAG) could improve performance,^50^^,^^51^^,^^52^^,^^53^ our sensitivity analysis does not support this. Few-shot learning improved structural compliance but did not enhance prediction accuracy, likely due to overly simplified examples in the prompts. Further investigation is needed to improve example-based prompts for better predictive performance. For RAG, the reduced performance observed is likely due to the knowledgebase used for indexing already being incorporated during the LLM’s training. Another finding highlights the importance of document indexing for knowledge retrieval; because RAG heavily relies on the retrieval steps, if relevant knowledge cannot be retrieved using an embedding similarity-based approach, then prediction performance may suffer.

From a practical perspective, considering that GPT-4 is almost 35 times more expensive than GPT-3.5 (according to the August 2023 billing policy in OpenAI, https://openai.com/api/pricing/), using more detailed prompts with smaller LLMs might be a more environmentally and economically friendly solution for certain tasks. Unfortunately, our study found that different prompts affected completeness and structural compliance in opposite ways, with no significant improvements in prediction accuracy. While some studies suggest that prompt influence is less dominant for certain tasks,^54^^,^^55^ we believe this remains an unresolved issue, especially because crafting efficient prompts for LLMs is still a challenge. We also emphasize that the cost of RAG experiments in this study is significantly higher due to the longer prompts incorporating documents from the priority-indexed knowledgebase. Future research should focus on strategic model selection based on specific needs and constraints to optimize both cost and computation speed.

### Conclusions

In this study, we conducted a comprehensive evaluation of the LLMs for phenotype-driven gene prioritization, a real-world application crucial in rare genetic disorder diagnosis. Even the best-performing model, GPT-4, still lags behind traditional bioinformatics tools in terms of generating accurate candidate gene prediction results. However, a clear trend of LLM performance increasing with model size is observed. Notably, LLMs’ ability to process free-text phenotypic descriptions is advantageous, although it may not achieve the same level of robustness as terminology-based input. These findings contribute to the ongoing discussion about integrating advanced LLMs into clinical genomic analysis workflows.

## Data and code availability

The datasets and code generated for this study are publicly available in the GitHub repository LLM-Gene-Prioritization

(https://github.com/stormliucong/LLM-Gene-Prioritization/tree/main), which includes all data files and source codes used in the analyses. Direct inquiries and requests for additional information can be addressed to the corresponding author. For more detailed information regarding the input dataset, please refer to https://github.com/stormliucong/LLM-Gene-Prioritization/blob/main/data/input/README.md.

## Acknowledgments

This study is supported by grants R01HG012655, R01LM012895, and R01HG013031 from the 10.13039/100000051National Human Genome Research Institute (10.13039/100000051NHGRI). We acknowledge Dr. Jingye Yang’s advice in using Llama2 locally.

## Declaration of interests

The authors declare no competing interests.

## Supplemental information

Tables S1 and S8–S11

Document S2. Article plus supplemental information
