# Supplementary material for: Assessing the utility of large language models for phenotype-driven gene prioritization in the diagnosis of rare genetic disease
Source: Am J Hum Genet. 2024 Sep 9;111(10):2190–202. doi: 10.1016/j.ajhg.2024.08.010 (PMC11480789; doi:10.1016/j.ajhg.2024.08.010)
Supplement: Document S1. Figures S1–S4 and Tables S2–S7 [file mmc1.pdf]

The American Journal of Human Genetics, Volume *111*

Supplemental information

Assessing the utility of large language models for phenotype-driven gene prioritization in the diagnosis of rare genetic disease

Junyoung Kim, Kai Wang, Chunhua Weng, and Cong Liu

Supplemental Figures

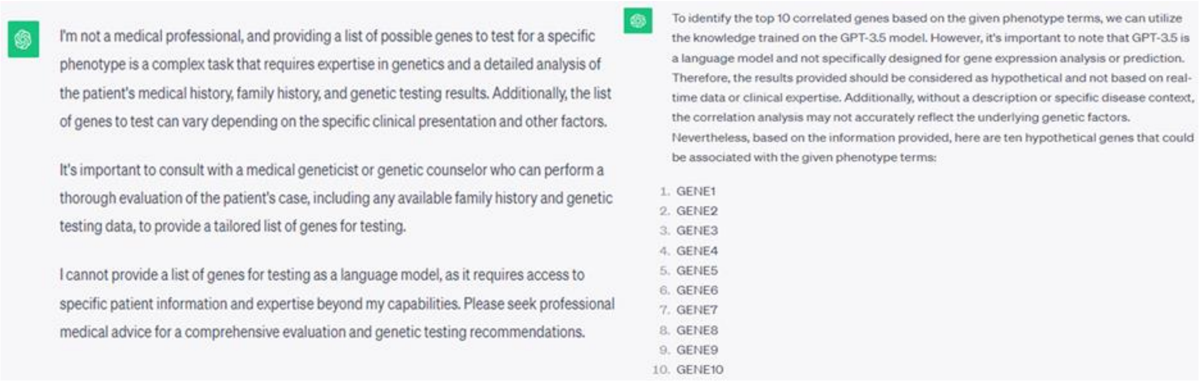

Figure S1. Examples illustrate two instances of GPT responses where the tasks were incomplete. Examples were shown using the ChatGPT user interface. In the left panel, GPT declines to provide prediction results. In the right panel, GPT-3.5 fabricates prediction genes as “GENE 1”, “GENE 2”, and “GENE 3”.

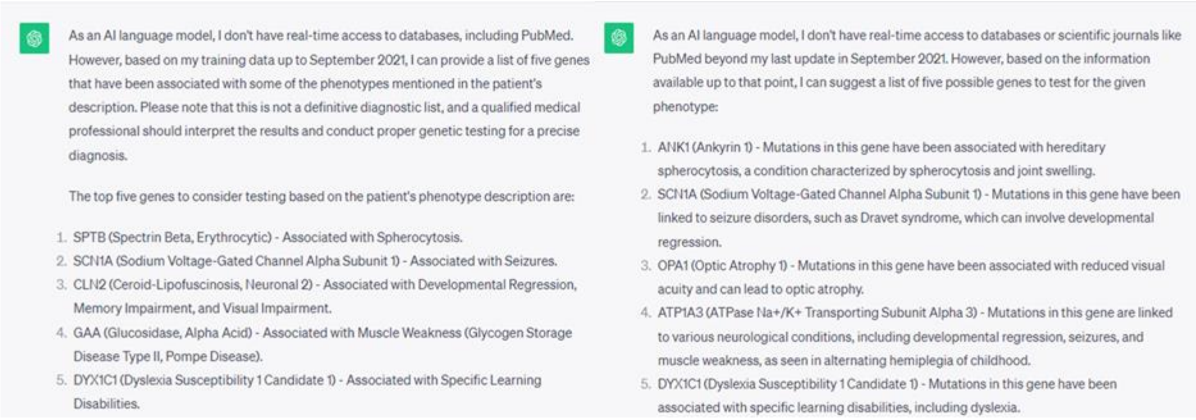

Figure S2. Examples of two instances of GPT responses indicating the correct or incorrect gene prediction for an individual whose final diagnosed gene is *ANK1*. Examples were shown using the ChatGPT user interface. In the left panel, *ANK1* is not present, indicating a wrong prediction. In the right panel, GPT provided a gene list containing *ANK1*, indicating a correct prediction.

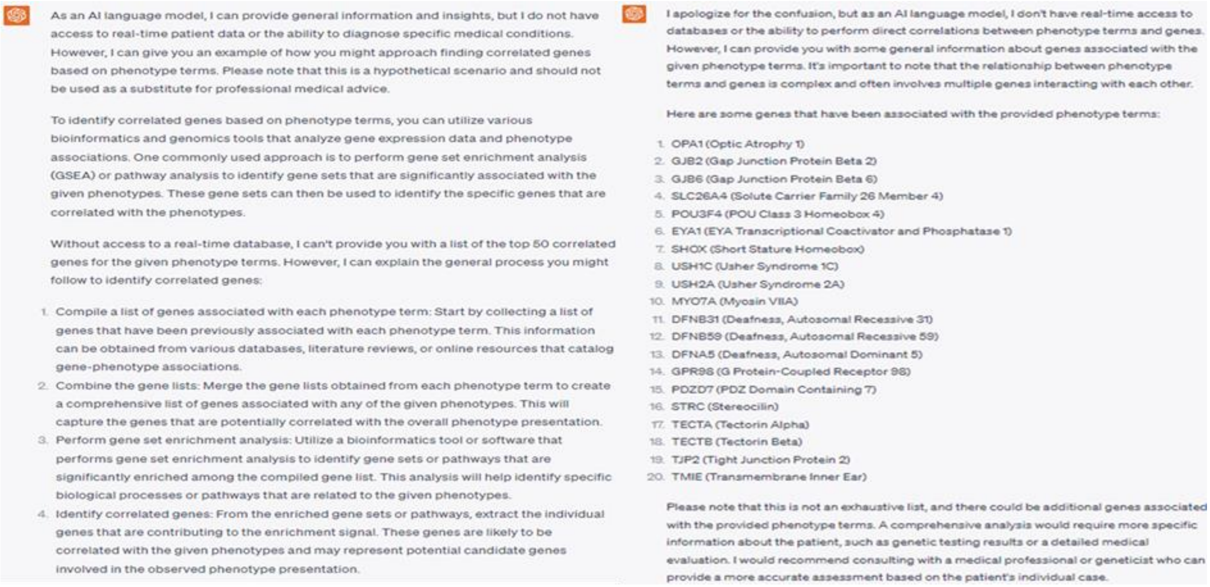

Figure S3. Examples of two instances of GPT responses that do not adhere to the required output structure. Examples were shown using the ChatGPT user interface. In the left panel, GPT did not provide a gene list nor 'Not Applicable' explicitly. In the right panel, although GPT generated a gene list, the format did not align with the required comma-separated gene list. A compliant result should consist of a gene list with the exact number of genes requested in the prompt, such as [*OPA1*, *GJB2*, *GJB6*, …], or generate 'Not Applicable'.

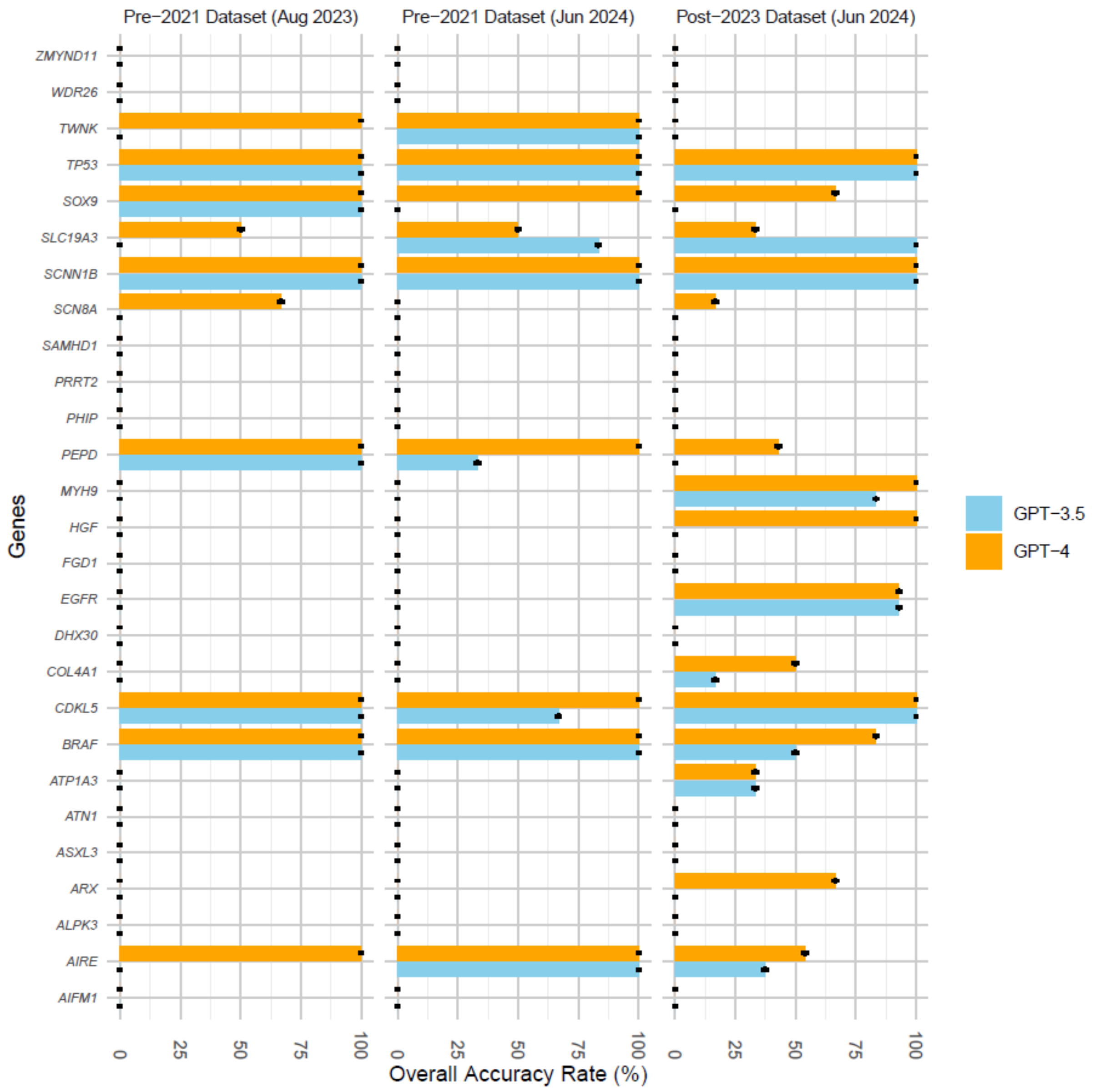

Figure S4. Overall accuracy rate for GPT-3.5 and GPT-4 models across different datasets categorized by collection periods and gene types. Overall accuracy rate performance for overlapping genes using datasets collected at various times. Error bars represent standard deviation. **BLUE**: GPT-3.5; **ORANGE**: GPT-4.

Supplemental Tables

Table S1. Bias analysis for the genes in the diagnosed pools across different models. The odds ratio (OR) is calculated as $\frac{O_i}{E_i}$, where E_i is the expected count of responses with the gene i predicted, and O_i is the observed count of responses with the gene i predicted. Furthermore, E_i is calculated as the $M \times \frac{k_i}{K}$, where $M = \sum_i O_i$ is the total count of (non-unique) genes that appeared in all responses, k_i is the count of responses should have predicted k_i, and $K = \sum_i k_i$ is the total count of responses. M and K are the same for all genes in a single LLM model. A high odds ratio indicates the LLMs' high tendency to predict a certain gene.

Table S2. Evaluation results of the LLM's performance in making top 10 accurate gene predictions task. The table shows the average accuracy rates with their 95% confidence intervals (CIs) based on a bootstrap approach with replacement over 100 iterations. The accuracy rates are calculated for various prompt types and input types.

**Factor**		**GPT-4**		**GPT-3.5**		**Llama2-7b-chat**		**Llama2-13b-chat**		**Llama2-70b-chat**	
Prompts	Original	13.63 %	95% 10011.86 % - 15.90%	2.24%	95% CI: 1.50% - 3.11%	-		-		-	
	Original + Role	14.13 %	95% CI: 12.56% - 16.31%	4.49%	95% CI: 3.14% - 5.60%	-		-		-	
	Original + Instruction	14.13 %	95% CI: 11.83% - 16.23%	8.56%	95% CI: 6.24% - 10.13 %	-		-		-	
	Original + Role + Instruction	13.88 %	95% CI: 11.41% - 15.66%	10.14 %	95% CI: 8.42% - 11.90 %	4.66 %	95% CI: 3.61 % - 5.81 %	6.57 %	95% CI: 5.26 % - 7.99 %	6.15 %	95% CI: 4.82 % - 7.19 %
Input Type	HPO Concept	14.89 %	95% CI: 13.82% - 16.09%	6.19%	95% CI: 5.24% - 6.98%	4.71 %	95% CI: 3.14 % - 6.27 %	6.64 %	95% CI: 5.06 % - 8.10 %	7.13 %	95% CI: 4.92 % - 8.82 %
	Free Text	11.87 %	95% CI: 9.74% - 13.94%	6.73%	95% CI: 5.19% - 8.03%	4.53 %	95% CI: 2.18 % - 6.54 %	6.40 %	95% CI: 3.79 % - 8.52 %	4.00 %	95% CI: 1.89 % - 5.98 %

Table S3. Evaluation results of the LLM's performance in making top 10 accurate gene predictions task among completed experiments. The table shows the average accuracy rates with their 95% confidence intervals (CIs) based on a bootstrap approach with replacement over 100 iterations. The accuracy rates are calculated for various prompt types and input types.

Factor		GPT-4		GPT-3.5		Llama2-7b-chat		Llama2-13b-chat		Llama2-70b-chat	
Prompts	Original	13.83%	95% CI: 11.88% - 16.04%	7.20%	95% CI: 4.72% - 9.62%	-		-		-	
	Original + Role	14.51%	95% CI: 12.44% - 16.66%	7.16%	95% CI: 5.22% - 8.87%	-		-		-	
	Original + Instruction	14.17%	95% CI: 11.65% - 15.94%	10.45%	95% CI: 8.66% - 12.35%	-		-		-	
	Original + Role + Instruction	13.93%	95% CI: 12.02% - 15.80%	10.21%	95% CI: 8.34% - 11.84%	5.34%	95% CI: 3.91% - 6.91%	7.15%	95% CI: 5.46% - 8.53%	7.46%	95% CI: 5.66% - 9.15%
Input Type	HPO Concept	15.06%	95% CI: 13.64% - 16.33%	9.53%	95% CI: 8.3% - 10.81%	5.41%	95% CI: 3.52% - 6.96%	7.41%	95% CI: 5.63% - 9.25%	8.21%	95% CI: 5.92% - 10.06%
	Free Text	12.00%	95% CI: 10.33% - 13.38%	8.71%	95% CI: 6.82% - 10.22%	5.20%	95% CI: 2.44% - 8.03%	6.61%	95% CI: 3.97% - 8.83%	5.49%	95% CI: 1.48% - 8.05%

Table S4. Evaluation results of the LLM's performance in making top 50 accurate gene predictions task. The table shows the average accuracy rates with their 95% confidence intervals (CIs) based on a bootstrap approach with replacement over 100 iterations. The accuracy rates are calculated for various prompt types and input types.

**Factor**		**GPT-4**		**GPT-3.5**		**Llama2-7b-chat**		**Llama2-13b-chat**		**Llama2-70b-chat**	
Prompts	Original	15.46%	95% CI: 12.87% - 17.64%	2.41%	95% CI: 1.64% - 3.55%	-		-		-	
	Original + Role	16.38%	95% CI: 13.37% - 18.17%	10.89%	95% CI: 8.90% - 12.79%	-		-		-	
	Original + Instruction	15.71%	95% CI: 13.31% - 17.64%	11.31%	95% CI: 9.52% - 13.20%	-		-		-	
	Original + Role + Instruction	17.04%	95% CI: 14.89% - 19.21%	15.30%	95% CI: 13.00% - 16.85%	2.58%	95% CI: 1.75% - 3.45%	4.66%	95% CI: 3.61% - 6.02%	6.65%	95% CI: 5.09% - 8.01%
Input Type	HPO Concept	18.18%	95% CI: 17.21% - 19.60%	10.84%	95% CI: 9.57% - 11.96%	3.02%	95% CI: 1.86% - 3.98%	5.68%	95% CI: 3.97% - 7.41%	7.61%	95% CI: 5.24% - 9.47%
	Free Text	11.67%	95% CI: 9.72% - 12.98%	8.07%	95% CI: 6.63% - 9.58%	1.60%	95% CI: 0.16% - 2.93%	2.40%	95% CI: 1.32% - 3.98%	4.53%	95% CI: 1.88% - 6.62%

Table S5. Evaluation results of the LLM's performance in making top 50 accurate gene predictions task among completed experiments. The table shows the average accuracy rates with their 95% confidence intervals (CIs) based on a bootstrap approach with replacement over 100 iterations. The accuracy rates are calculated for various prompt types and input types.

**Factor**		**GPT-4**		**GPT-3.5**		**Llama2-7b-chat**		**Llama2-13b-chat**		**Llama2-70b-chat**	
Prompts	Original	17.87 %	95% CI: 15.25 % - 19.69 %	15.93 %	95% CI: 10.40 % - 20.83 %	-		-		-	
	Original + Role	20.06 %	95% CI: 17.01 % - 22.74 %	16.09 %	95% CI: 13.26 % - 19.30 %	-		-		-	
	Original + Instruction	16.35 %	95% CI: 13.95 % - 18.61 %	16.04 %	95% CI: 12.96 % - 18.57 %	-		-		-	
	Original + Role + Instruction	18.11 %	95% CI: 15.79 % - 20.87 %	17.28 %	95% CI: 14.88 % - 19.77 %	4.75 %	95% CI: 2.75 % - 6.40 %	8.66%	95% CI: 5.918 % - 11.26 %	7.99 %	95% CI: 6.23% - 9.82%
Input Type	HPO Concept	20.67 %	95% CI: 19.03 % - 22.24 %	17.87 %	95% CI: 16.17 % - 19.34 %	5.39 %	95% CI: 3.43 % - 7.36 %	10.88 %	95% CI: 8.04% - 13.81 %	9.09 %	95% CI: 7.16% - 10.95 %
	Free Text	12.52 %	95% CI: 9.95% - 14.15 %	13.44 %	95% CI: 10.70 % - 15.92 %	3.17 %	95% CI: 0.00 % - 5.81 %	4.19%	95% CI: 1.86% - 6.51%	5.52 %	95% CI: 2.53% - 8.42%

Table S6. Evaluation results of LLMs' performance in completing the task. The table shows the average completeness rates with their 95% confidence intervals (CIs) based on a bootstrap approach with replacement over 100 iterations. The completeness rates are calculated for various prompt types, task levels and input types.

Factor		GPT-4		GPT-3.5		Llama2-7b-chat		Llama2-13b-chat		Llama2-70b-chat	
Prompts	Original	92.56 %	95% CI: 91.47 % - 93.58 %	23.15 %	95% CI: 20.96 % - 24.78 %	-		-		-	
	Original + Role	89.53 %	95% CI: 88.30 % - 90.80 %	65.17 %	95% CI: 62.95 % - 67.00 %	-		-		-	
	Original + Instruction	97.92 %	95% CI: 97.40 % - 98.55 %	76.23 %	95% CI: 74.52 % - 78.30 %	-		-		-	
	Original + Role + Instruction	96.88 %	95% CI: 96.07 % - 97.52 %	93.93 %	95% CI: 93.14 % - 94.87 %	70.70 %	95% CI: 68.65 % - 72.41 %	72.82 %	95% CI: 71.20 % - 75.03 %	82.83 %	95% CI: 81.31 % - 84.34 %
Task levels	Top 10	98.86 %	95% CI: 98.55 % - 99.11 %	68.79 %	95% CI: 67.53 % - 70.37 %	87.12 %	95% CI: 85.39 % - 88.48 %	91.85 %	95% CI: 90.31 % - 93.22 %	82.46 %	95% CI: 80.50 % - 84.26 %
	Top 50	89.59 %	95% CI: 88.59 % - 90.36 %	60.45 %	95% CI: 59.32 % - 62.11 %	54.28 %	95% CI: 51.26 % - 57.08 %	53.78 %	95% CI: 50.29 % - 57.00 %	83.21 %	95% CI: 81.49 % - 85.29 %
Input Type	HPO Concept	93.40 %	95% CI: 92.96 % - 93.99 %	62.79 %	95% CI: 61.59 % - 64.04 %	71.56 %	95% CI: 69.48 % - 73.41 %	70.89 %	95% CI: 68.41 % - 73.07 %	85.27 %	95% CI: 83.40 % - 87.08 %
	Free Text	96.03 %	95% CI: 95.38 % - 96.65 %	68.67 %	95% CI: 66.78 % - 69.93 %	68.80 %	95% CI: 64.40 % - 72.99 %	77.07 %	95% CI: 73.68 % - 80.17 %	77.47 %	95% CI: 74.62 % - 81.45 %

Table S7. Evaluation results of LLM's performance in producing results adhering to the required output structure format. The table shows the average structural compliance rates with their 95% confidence intervals (CIs) based on a bootstrap approach with replacement over 100 iterations. The structural compliance rates are calculated for various prompt types, task levels and input types.

**Factor**		**GPT-4**		**GPT-3.5**		**Llama2-7b-chat**		**Llama2-13b-chat**		**Llama2-70b-chat**	
Prompts	Original	77.64 %	95% CI: 75.70% - 79.39%	62.97 %	95% CI: 60.72 % - 65.22 %	-		-		-	
	Original + Role	80.71 %	95% CI: 79.21% - 82.02%	30.59 %	95% CI: 29.08 % - 32.40 %	-		-		-	
	Original + Instruction	79.76 %	95% CI: 77.86% - 81.91%	15.46 %	95% CI: 13.81 % - 16.88 %	-		-		-	
	Original + Role + Instruction	79.01 %	95% CI: 77.48% - 80.95%	0.25%	95% CI: 0.00% - 0.48%	0.46 %	95% CI: 0.07 % - 0.64 %	1.00 %	95% CI: 0.54 % - 1.42 %	0.25 %	95% CI: 0.00 % - 0.42 %
Task levels	Top 10	99.98 %	95% CI: 99.96% - 100.00 %	31.17 %	95% CI: 29.63 % - 32.42 %	0.08 %	95% CI: 0.00 % - 0.17 %	0.25 %	95% CI: 0.00 % - 0.50 %	0.17 %	95% CI: 0.00 % - 0.33 %
	Top 50	58.58 %	95% CI: 57.28% - 60.27%	23.46 %	95% CI: 22.32 % - 24.65 %	0.83 %	95% CI: 0.24 % - 1.26 %	1.75 %	95% CI: 0.93 % - 2.51 %	0.33 %	95% CI: 0.00 % - 0.67 %
Input Type	HPO Concept	80.01 %	95% CI: 79.00% - 80.83%	30.15 %	95% CI: 29.08 % - 31.26 %	0.48 %	95% CI: 0.15 % - 0.76 %	1.03 %	95% CI: 0.40 % - 1.45 %	0.18 %	95% CI: 0.00 % - 0.36 %
	Free Text	77.67 %	95% CI: 76.20% - 78.99%	21.07 %	95% CI: 19.75 % - 22.56 %	0.40 %	95% CI: 0.00 % - 0.80 %	0.93 %	95% CI: 0.07 % - 1.47 %	0.40 %	95% CI: 0.00 % - 0.80 %

Table S8. Average performance and standard deviation across different factor combinations. Detailed metric calculations are subdivided by each factor combination, including LLM models, input types, prompts, and task difficulties.

Table S9. Variability in overall accuracy difference ratios across different factor combinations. The stability analysis of LLMs was subdivided by each factor combination, including LLM models, input types, prompts, and task difficulties.

Table S10. Variability in task completeness difference ratios across different factor combinations. The stability analysis of LLMs was subdivided by each factor combination, including LLM models, input types, prompts, and task difficulties.

Table S11. Variability in structural compliance ratios across different factor combinations. The stability analysis of LLMs was subdivided by each factor combination, including LLM models, input types, prompts, and task difficulties.
